# Race and Sex‐ specific Association of Sleepiness Scores and SIMOA assessed plasma AD biomarkers in Cognitively Normal Non‐Sleepy Older Adults

**DOI:** 10.1002/alz70856_107095

**Published:** 2026-01-08

**Authors:** Anthony Q Briggs, Joshua L Gills, Mark A Bernard, Rachel A McCray, Elena Valkanova, Mobeena Ghuman, Allal Boutajangout, Ludovic Debure, Girardin A Jean‐Louis, Arjun V. Masurkar, Ricardo S. Osorio, Omonigho M Bubu

**Affiliations:** ^1^ NYU Alzheimer's Disease Research Center, New York, NY, USA; ^2^ NYU Grossman School of Medicine, New York, NY, USA; ^3^ NYU Langone Health Center for Cognitive Neurology, New York, NY, USA; ^4^ Center for Cognitive Neurology, NYU Grossman School of Medicine, New York, NY, USA; ^5^ University of South Florida, Tampa, FL, USA; ^6^ Center for Cognitive Neurology, New York, NY, USA; ^7^ NYU School of Medicine, New York, NY, USA; ^8^ Center for Cognitive Neurology, New York University Langone Health, New York, NY, USA; ^9^ University of Miami Miller School of Medicine, Miami, FL, USA; ^10^ New York University Langone Health, New York University Grossman School of Medicine, New York, NY, USA; ^11^ Alzheimer's Disease Research Center, New York University Langone Health, New York, NY, USA; ^12^ New York University Grossman School of Medicine, New York, NY, USA; ^13^ Health Brain Aging and Sleep Center, New York, NY, USA; ^14^ Center for Sleep and Brain Health, Department of Psychiatry, NYU Langone Health, New York, NY, USA; ^15^ Nathan S. Kline Institute, Orangeburg, NY, USA

## Abstract

**Background:**

Excessive daytime sleepiness (EDS) is associated with poor cognitive performance and increased Alzheimer's Disease (AD) risk. However, the association between sleepiness scores in non‐ sleepy individuals and plasma AD biomarkers remains under‐explored. We examined the association between sleepiness scores and plasma AD biomarkers in a diverse sample of cognitively normal community dwelling non‐ sleepy older adults and studied for race and sex differences of this association.

**Method:**

We conducted a cross‐sectional analysis of 186 cognitively normal, community‐ dwelling older adults (103 whites, 83 blacks) from various NYU studies on sleep, aging, and memory. Excessive daytime sleepiness (EDS) was assessed using the Epworth Sleepiness Scale (ESS). Plasma AD biomarkers (Aß‐42, Aß, *p*‐tau, NfL, Total Tau, GFAP) were measured using SIMOA. Linear mixed effects models controlled for age, sex, race, cognitive status, and education, examining ESS association with plasma markers and interactions between ESS* race and ESS*sex.

**Result:**

The study participants had an average of 72.7±6.5y, and 16.6± 2.2y of education. 68.8% of participants female, Mean ± SD for ESS was 4.2± 3.7 (≥ 11 suggest EDS), and 407.0 ± 85.6 minutes for total sleep time. Independently, sleepiness scores were not associated with the levels of any Plasma AD biomarkers. However, in the interaction model for GFAP levels, race ESS, and Race*ESS interaction were significant (ß[Race*ESS] = 9.67[2.99, 16.35]; *p* <0.05). With each unit increase in sleepiness scores, Blacks were more likely to have higher GFAP levels. Furthermore, Race only had an independent effect on pTau 181/Aß42 levels(ß[Race] =0.059 [‐0.117, ‐0.001] with Blacks having lower levels. Sex only had an independent effect on GFAP, Aß42/Aß40, and pTau181/Aß42, levels (ß[Sex]=27.55 [2.51, 52.58]; ß [Sex] = ‐0.06 [‐0.11, ‐0.01]; *p* < ) 0.05, respectively), with females having higher levels of GFAP, and Aß42/Aß40, but lower pTau181/Aß42 levels.

**Conclusion:**

In this non‐sleepy, cognitively healthy diverse sample of community dwelling older adults, sleepiness scores were not associated with any Plasma AD biomarkers. However, Race‐ specific effects of this association were observed with markers of inflammation, suggesting that neuronal inflammation may be related to EDS symptoms with a notable effect among Blacks.

